# Spectrofluorometric investigations on the solvent effects on the photocyclization reaction of diclofenac

**DOI:** 10.1016/j.heliyon.2023.e20767

**Published:** 2023-10-11

**Authors:** Abdulilah Dawoud Bani-Yaseen, Raed M. Al-Zoubi, Mohanad Shkoor

**Affiliations:** a Department of Chemistry & Earth Sciences, Faculty of Arts & Science, Qatar University, P.O. Box: 2713, Doha, Qatar; b Department of Biomedical Sciences, College of Health Sciences, QU-Health, Qatar University, Doha, 2713, Qatar; c Surgical Research Section, Department of Surgery, Hamad Medical Corporation, Doha, Qatar; d Department of Chemistry, Jordan University of Science and Technology, P.O.Box 3030, Irbid, 22110, Jordan

**Keywords:** Diclofenac, Photoconversion, Fluorescence, Solvent effects, Solvatochromic models

## Abstract

The solvent effects on the photochemical conversion rate of the photosensitizing drug diclofenac (DCF) were investigated using steady-state fluorescence spectroscopy. The spectral information obtained for the photochemical reaction of DCF in a set of neat solvents demonstrates that the photoconversion reaction rate of DCF is not only medium polarity dependent but also hydrogen-bonding dependent. The solvent effects were qualitatively and quantitatively assessed employing various solvatochromic models, including multi-parameter linear regression analysis (MLRA). Interestingly, the MLRA results (R = 0.99) revealed that the photoconversion rate increases with increasing solvent polarizability (π*) and H-bond donor capability (*α*), whereas the rate decreases with increasing hydrogen-bond acceptor capability (β). However, predominant effect of the solvent acidity compared to basicity and polarizability was observed. A hypothesis rationalizing the effects of H-bonding and medium polarity on DCF photoconversion reaction is presented and discussed.

## 1 Introduction

Recent interest in photosensitive materials, notably pharmaceuticals, has increased significantly [[1], [2], [3], [4], [5], [6], [7], [8], [9], [10]]. Due to the potential adverse effects of light exposure, including loss of therapeutic efficacy and, more importantly, the induction of unanticipated clinical states, the photosensitivity of drug formulations is being investigated [1,4,6]. Therefore, it is necessary to understand the photosensitivity and photoreactivity of various pharmaceutical substances for their proper administration. Importantly, photosensitive pharmaceuticals respond differently to light, including photodecomposition and particular photochemical reactions like photocyclization [[11], [12], [13]]. Diclofenac (DCF) (2-[2′,6'-(dichlorophenyl)amino](phenyl)acetic acid) is one of the pharmaceutical entities that is highly photosensitive with potentially hazardous properties. DCF is one of the most commonly prescribed nonsteroidal anti-inflammatory drugs (NSAIDs) for the treatment of pain and inflammation associated with a variety of rheumatic and non-rheumatic diseases. Because DCF is extensively used throughout the globe, considerable concern should be expressed not only regarding its potentially hazardous properties but also regarding its photochemical byproducts [14]. The vast majority of published research on the photochemical reaction of DCF has focused on elucidating the mechanism of its conversion into various photoproducts and their biological effects without considering the role of medium polarity and hydrogen bonding ability in its photochemical transformations [[12], [13], [14], [15], [16]].

Solvents and medium can have a profound effect on physical and chemical phenomena, including chemical reactions, and are crucial when investigating the mechanisms of different processes [[17], [18], [19], [20], [21], [22], [23], [24], [25], [26]]. Hydrogen bonding is an important aspect of solvent effects in photochemical reactions [[27], [28], [29], [30]]. The photochemical oxidation of anisole, for instance, could be efficiently driven by specific solvent/solute interactions, with the increase in reaction efficiency explained by the solvent's hydrogen-bond donating capabilities [27]. In addition, the significance of hydrogen bonding between a substrate and free radicals in enhancing the selectivity of hydrogen atom abstraction along with an explanation of the consequences of hydrogen-bonded complexes were highlighted [28]. Understanding how medium and temperature affect the photo-physicochemical characteristics and photoreactivity of photosensitive pharmaceuticals like DCF is important since the degree of phototoxicity in biological systems may be medium- and temperature-dependent.

**Fig. 1:**
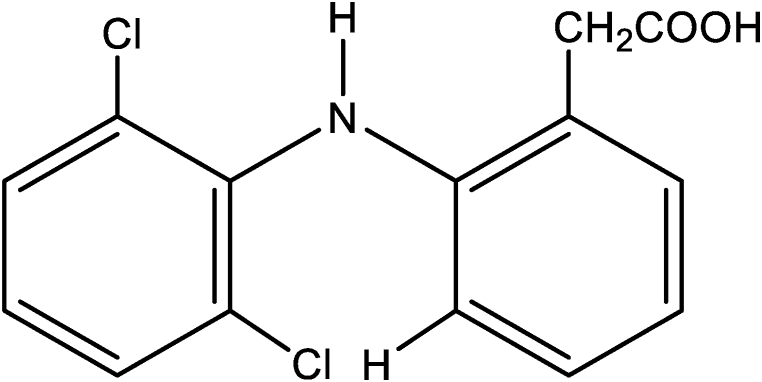
Chemical structure of DCF.

Several reports detail how the medium influences DCF photoconversion reactions catalyzed and uncatalyzed. Physical and chemical factors, as well as the medium matrix and other solution components, were shown to have an impact on the photoconversion rate [[31], [32], [33], [34], [35], [36], [37]]. As a result, there is a significant need for systematic qualitative and quantitative analyses of such effects. This can be accomplished by combining various solvatochromic models with specific spectral properties that address solvent/solute specific interactions, such as hydrogen bonding. In this study, we examined the medium effects on the photoconversion reaction of DCF, with a focus on polarity and solvent/solute specific interactions (hydrogen bonding) as significant factors influencing reaction rate. Fig. 1 displays the chemical structure of DCF.

In this study, we present a quantitative examination of the solvent's effects on the rate of the photochemical reaction of DCF utilizing steady-state fluorescence spectroscopy. The steady-state fluorescence spectra of DCF were measured as a function of temperature, solvent, and irradiation time, and the data were kinetically correlated and interpreted using various solvatochromic parameters such as orientation polarizability (Δƒ), polarity index (E_T_(30)), and multi-parameter linear regression analysis (MLRA), taking into account not only the solvent polarity but also its hydrogen bonding capability.

## 2 Experimental

### 2.1 Materials

DCF and carbazole were obtained from Sigma-Aldrich, and solvents (spectroscopic grade) were purchased from different commercial suppliers. All materials were used as received. Aqueous solutions were prepared using Milli-Q ultrapure water (Millipore, 18 MΩ).

### 2.2 Instrumentation and measurements

Absorption spectra were obtained using a Cary 100 Conc UV–Vis spectrophotometer (Agilent). The steady-state fluorescence spectra of DCF were measured using an RF-5301PC spectrofluorometer (Shimadzu) equipped with a 150 W xenon lamp. Fluorescence measurements were obtained in a 1.0-cm quartz cuvette with a constant-temperature cell holder connected to a water circulator (Shimadzu, Japan). The excitation and emission bandwidths were set to 3 and 5 nm, respectively, with an excitation wavelength (λ_ex_) of 240 nm. A 1 × 10^−3^ M stock solution of DCF was prepared in methanol. Photokinetics experiments were conducted with 1.3 × 10^−6^ M solutions of DCF in Milli-Q ultrapure water and different solvents. The 1.3 × 10^−6^ M solutions of DCF in different solvents were prepared by withdrawing an appropriate volume of DCF stock solution, evaporating the methanol, and re-dissolving the drug residue in an appropriate volume of the target solvent to yield a 1.3 × 10^−6^ M DCF solution.

### 2.3 Kinetics experiments

Photo-irradiation was performed on a 1.3 × 10^−6^ M solution of DCF using a 1.0 cm quartz cuvette housed in an external constant-temperature cell holder. The progress of the photoreaction was monitored by measuring the fluorescence spectra after various periods of irradiation at 254 nm using a 6 W lamp located approximately 5 cm from the cell holder with continuous stirring.

## 3 Results and discussion

**Scheme 1:**
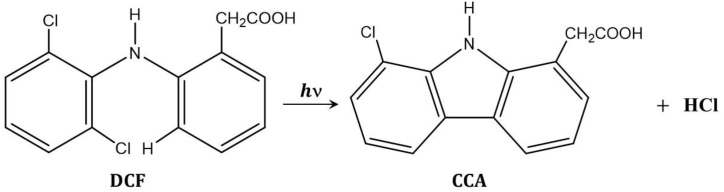
Photoconversion reactions of DCF to CCA.

**Fig. 2:**
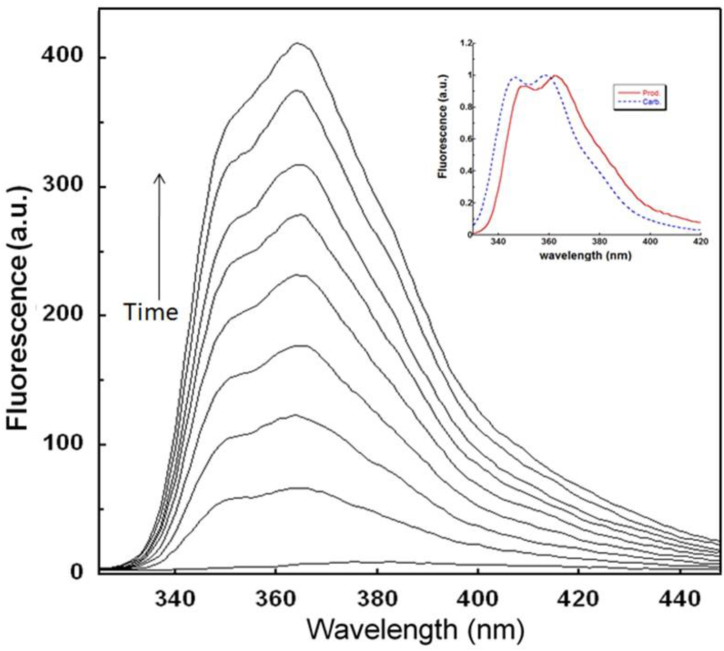
Fluorescence spectra obtained over 0−5 min for the UV irradiation of DCF in aqueous solution: [DCF]_o_ = 1.3 × 10^−6^ M. The arrow indicates the growth in product fluorescence with increasing irradiation time. Inset: fluorescence spectra of carbazole and DFC obtained after irradiation.

Photoinduced fluorescence spectroscopy is a very sensitive technique that is widely used for monitoring chemical reactions, especially when the analyte is present at a low concentration. In principle, the process involves the photochemical conversion of a poorly fluorescent reactant into a highly fluorescent product, where monitoring the change in the fluorescence intensity of the latter permits examining the kinetics of the photochemical reaction [[38], [39], [40], [41], [42]]. DCF exhibits poor fluorescence properties under normal conditions, such as those given in the experimental section [[13], [14], [15], [16]]; however, the fluorescence spectrum observed after exposure to UV irradiation clearly demonstrates the formation of highly fluorescent products, particularly chlorocarbazole acetic acid (CCA), via the photoconversion pathway as depicted in Scheme 1. Nevertheless, the photoreaction of DCF can be adequately described by a pseudo-first-order kinetics model:

(1) $$\nu =\frac{d\left[CCAs\right]}{dt}=k_{\text{app}}\left[\text{DCF}\right]$$

where *k*_app_ is the apparent rate constant of the photocyclization reaction, [DCF] is the DCF concentration, and [CCAs] is the total concentration of all fluorescent carbazole derivatives.

generated from the photochemical conversion of DCF. As expressed in Eq. (1), the rate of the reaction can be calculated using the change in fluorescence intensity (integrated) as a function of time. The steady-state fluorescence spectra of a 1.3 × 10^−6^ M aqueous DCF solution after different irradiation times (λ_irr_ = 254 nm) are depicted in Fig. 2. The fluorescence spectrum displays two bands around 350 and 366 nm, similar to those observed for carbazole derivatives such as chlorocarbazole and consistent with previous reports [[12], [13], [14], [15], [16]]. In contrast, the fluorescence spectrum of the product observed after the UV irradiation of DCF displays a minor solvent polarity dependence (data not shown); similar two-band structures were observed in all tested solvents with negligible shifts in the position of $\lambda_{Fluo}^{\max}$.

**Fig. 3:**
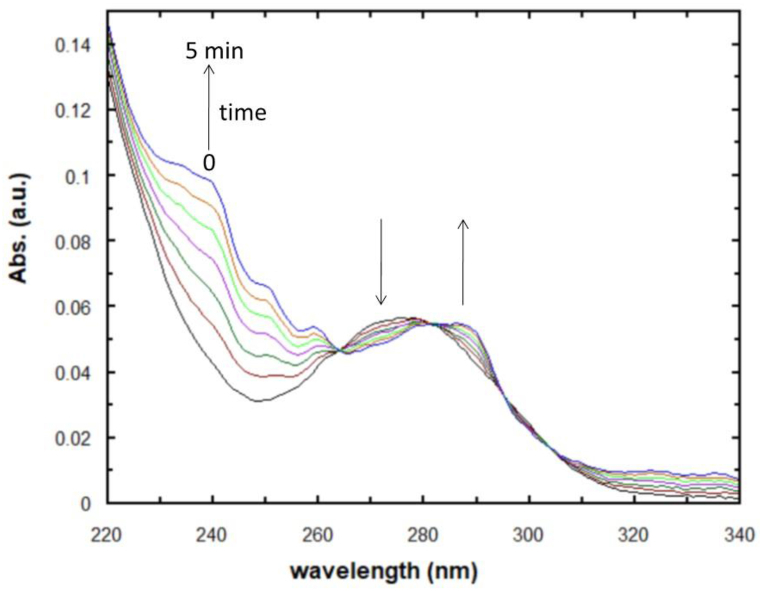
Absorption spectra obtained over 0−5 min for the UV irradiation of DCF in aqueous solution: [DCF]_o_ = 1.3 × 10^−6^ M. The arrows indicate the growth and reduction of absorption bands with increasing irradiation time.

In addition, the stability of the fluorescence signal after the irradiation period was investigated via repeated measurement of the fluorescence spectrum of the same solution over 48 h. The signal consistency varied by less than 1 %, indicating the formation of a highly stable photoproduct as would be expected for a carbazole and its derivatives under the given experimental conditions. It should be noted that the working solution displayed an initial.

absorbance of ≤0.05 for the main absorption band (λmax = 287 nm), which permitted the fluorescence measurements without interference. On the other hand, the absorption spectra were measured for the same aqueous solution of DCF after different irradiation times, as displayed in Fig. 3. In this case, the obtained spectra display four isosbestic points that indicate the occurrence of the DCF photoconversion reaction, where the growth of bands at approximately 240, 287, and 330 nm is consistent with the observations from the product fluorescence spectra in terms of the formation of carbazole derivatives.

**Fig. 4:**
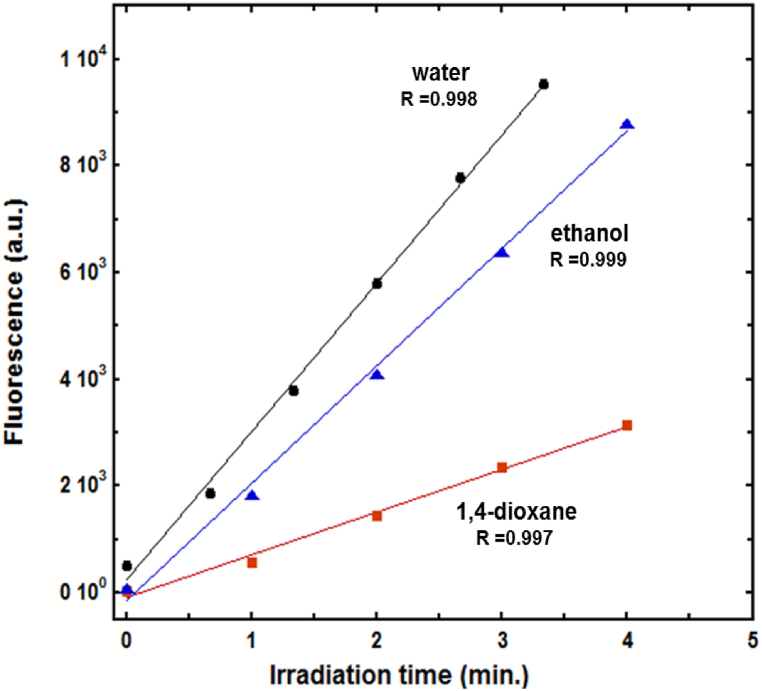
Variation in product fluorescence intensity as a function of irradiation time in selected solvents. [DCF]_o_ = 1.3 × 10^−6^ M.

Given the fluorescence behavior of the DCF photocyclization products, the reaction was kinetically monitored using steady-state fluorescence spectroscopy. Fig. 4 displays the variation of the photoproduct total fluorescence intensity as a function of irradiation time in selected solvents, namely water, ethanol, dichloromethane, and 1,4-dioxane. In water, the photoproduct fluorescence intensity starts to display a steady signal after approximately 6 min, whereas longer times are needed to achieve a steady signal in solvents of lower polarity. This can be attributed to the fact that the absorption peaks of the photoproducts overlap with those of the reactants, which in turn retards accurate estimation of the concentration or absorbance of the material of interest. Thus, fluorescence spectroscopy can yield more accurate data for the photochemical reaction rate of DCF. Hence, the kinetics data was employed to determine the reaction rate. For simplicity, the initial velocity (*ν*_0_) technique was employed for estimating the reaction rate, which was specified as the slope of the fluorescence intensity (*F*_int_) vs. time curve employing the pseudo-first-order kinetics expression in Eq. (1). Importantly, no attempts were made to quantify the quantum yield of the DCF photocyclization. It was recently reported that the photoreaction of DCF proceeds with a quantum yield that is independent of the wavelength of the UV irradiation [37,43], where the experimental procedure used an excitation wavelength of 254 nm under a low-pressure Hg lamp. Hence, as the main objective of this work is to investigate the effects of solvent on the rate of DCF photoconversion, no attempts were made to perform the phototransformation process at other wavelengths or obtain actinometric measurements for quantum yield estimation.

It is crucial to quantitatively analyze and explain the impact of different solvent characteristics in order to fully understand how solvents affect the mechanisms of photoconversion reactions. This analysis goes beyond just considering solvent polarity, it includes other important factors like specific interactions between the solvent and solute, such as their ability to form hydrogen bonds. The solvent parameters of interest, along with the obtained parameters of the DCF photochemical reaction, are listed in Table 1. As can be noted from Table 1, the fastest reaction is observed in a neat aqueous solution with a rate of 2911 a.u.·min^−1^. However, the rate decreases with decreasing solvent polarity: e.g., a rate of ∼800 a.u. min^−1^ is observed in 1,4-dioxane. The first attempt toward quantifying the solvent effects on the DCF photoconversion was conducted by applying the Lippert–Mataga technique. In principle, the dependence of a measurable physical parameter on solvent polarity is commonly presented in terms of solvent polarizability (Δ*f*) and an empirical polarity parameter, E_T_(30) [[44], [45], [46], [47]]. However, poor and inconclusive correlations were obtained upon plotting the photochemical reaction rate (*ν*) as a function of the Δ*f* and E_T_(30) values of different solvents (Fig. S1). Interestingly, the multiparameter linear regression analysis (MLRA) of Kamlet–Taft, a linear solvation energy relationship approach, takes into consideration various solvatochromic parameters and can generally offer an effective method by considering the dual influence of solvent polarity and hydrogen bonding [[46], [47], [48]]. This approach is presented as:

(2) $$Y=Y_{o}+a\alpha +b\beta +s\pi^{*}$$

where, *Y* and *Y*_o_ are measurable physical parameters in the presence and absence of solvent, respectively; π* is the solvent dipolarity/polarizability; *α* and *β* are the solvent hydrogen-bond donor (acidity) and acceptor (basicity) capabilities, respectively; and *s*, *a*, and *b* are independent coefficients that are obtained by the MLR data analysis, which indicate the relative contribution.

**Table 1:** Kinetics parameters of DCF in different solvents along with their physical parameters at 298 (±1) K.

solvent	Δ*f*	E_T_(30)	*α*	*ß*	π*	Rate (a.u. min^−1^)
*Polar protic*						
Water	0.3201	63.1	1.17	0.47	1.09	2911
Ethanol	0.2890	51.9	0.86	0.75	0.54	2235
Isopropanol	0.2764	48.4	0.76	0.84	0.48	2276
*Polar aprotic*						
CCl_2_H_2_	0.2184	40.7	0.13	0.10	0.82	1850
Ethyl Acetate	0.1998	38.1	0	0.45	0.55	513
*Nonpolar*						
1,4-Dioxane	0.0216	36.0	0	0.37	0.55	782
Hexane	0.0010	31.0	0	0	0	665

Abbreviations: Δƒ: orientation polarizability; E_T_(30): surface polarity; π*: dielectric effect of solvent; *β*: hydrogen-bond acceptor ability; *α*: hydrogen-bond donor ability.

for each respective parameter of the solvents. MLRA was performed using the DCF photochemical conversion rates (*ν*_app_) measured for all examined solvents. By considering these results, the following solvent-dependent linear regression equation for the rate of the DCF photochemical reaction in different solvents can be written:

(3) $$\nu_{app}={\nu_{app}}_{o}+\left(1731\;\pm \;532\right)\alpha -\left(425\;\pm \;745\right)\beta +{\left(564\;\pm \;615\right)\pi }^{*};\;R=0.950$$

**Fig. 5:**
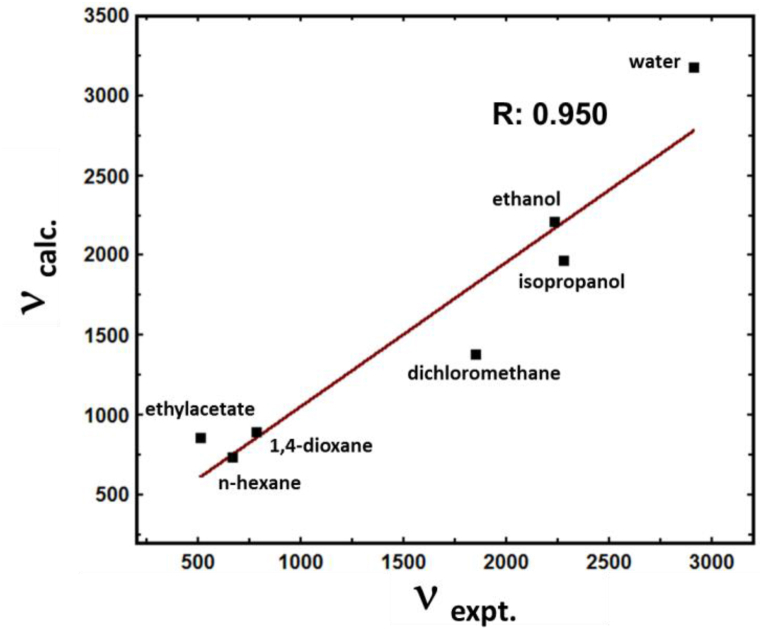
Correlation between the experimental and calculated rates of the DCF photoconversion; *ν*_calc_ was calculated using Eq. (3).

Inspecting Eq. (3), one must notice that the solvent's hydrogen-bond-accepting capability exhibits the potential to dominantly increase the rate of DCF photocyclization, whereas hydrogen-bond-donating capability retards the photoconversion, i.e., the negative sign implies destabilization. Importantly, then, solvent polarity is not the only determinant during the process of DCF photocyclization; hydrogen bonding is another important factor that must be considered. To test its validity and precision, Eq. (3) was employed to calculate the expected values of the reaction rates in different solvents. The correlation between the experimental (*ν*_expt._) and calculated (*ν*_calc._) values is depicted in Fig. 5. Clearly, Eq. (3) affords a good correlation (R = 0.950) between the calculated and experimental rates of the DCF photochemical reaction in solvents of different polarities and hydrogen bonding abilities. Thus, it is essential to consider hydrogen bonding as a decisive factor in the mechanistic pathway of DCF photoconversion. Our findings are in agreement with reported work concerning the effects of hydrogen bonding on hydrogen abstraction and, correspondingly, on the efficiency of photochemical reactions [29,49].

The comparable coefficients obtained for *α* and *β* compared with those obtained for π* imply that the hydrogen-bonding capability of the solvent in which the DCF photochemical reaction occurs contributes significantly to the apparent rate of the process. Yet, one can notice that the values of relative uncertainty obtained for β and π are high to be considered decisive factors in the rate of photocyclization of DCF compared to *α*. Accordingly, considering the opposite effects of solvent polarity vs. basicity and the high values of their corresponding relative uncertainty, we may hypothesize that their combined effects can be cancelled, and consequently, the solvent acidity can be considered as the dominant and key factor that controls the rate of the photocyclization of DCF. In this contents, attempts were made to determine a direct correlation between the reaction rate and *α*, *β*, and π* individually. From the results displayed in Fig. S2, a good correlation can be noted between the reaction rate and the solvent acidity (R = 0.930), whereas poor correlations are observed for the solvent basicity and polarizability. Nevertheless, comparing these results with those obtained from the MLRA (R = 0.950), one can infer the relative importance of considering these factors in combination rather than individually.

Various research has been conducted to examine various mechanistic aspects of the photoconversion of DCF, and alternative pathways have been proposed, including non-cyclization pathways and further photoconversions of the carbazole photoproducts [12,[14], [15], [16]]. However, it is essential to clarify that these alternative mechanisms were proposed based on different photochemical reaction conditions, which also postulated different intermediates. This suggests that it will be challenging to determine a single mechanism for the photoconversion of DCF. Nevertheless, the photoconversion of DCF proceeds via a multistep pathway to yield various carbazole derivatives; this pathway can generate different intermediates that lead to the corresponding photoproducts, where a major carbazole derivative is formed during the early stages of photochemical conversion. Thus, the intense and observable fluorescence properties of these carbazole derivatives facilitate the use of fluorescence spectroscopy to elucidate the factors that control the kinetics of the pathway through which DCF is converted into carbazole products, regardless of further conversions. Hence, neither further conversions nor other pathways will be considered in this discussion.

Moreover, with respect to the chemical structure of DCF, one can notice that it compiles various characteristics of an aryl halide (Ar-X) as well as diphenylamine (DPA). For such Ar-X, it can undergo a photocyclization reaction where a photo-dehalogenation would be highly expected before the photocyclization process [50,51]. In principle, Ar-X is capable of undergoing intramolecular photoreactions via two distinct processes, namely hemolysis and electron transfer. Both of these methods entail an aryl radical (Ar^●^) as an intermediate [[50], [51], [52], [53]]. On the other hand, for DPA, can undergo multi-step photocyclization reactions that involve different intermediate stages, ultimately resulting in the formation of photo-products containing carbazole. Accordingly, taking into account the characteristics that are to be anticipated from DCF in its capacity as a derivative of both DPA and Ar-X, it is reasonable to hypothesize that DCF goes through a photoconversion route and subsequent cyclization process that are analogous to those of DPA and Ar-X. Hitherto, several studies have probed the pathway through which the conversion of such compounds occurs [10,50,51,[54], [55], [56]]. However, it is crucial to recognize that all proposed mechanisms emphasize that the overall rate of the process is governed by the rate at which a small set of intermediates transform into one another. According to the findings of Rahn et al. , the most critical factors in the process of photocyclization are the substituent present on the amine and the participation of a triplet excited-state intermediate [51]. However, despite the fact that Rahn et al.'s pathway is typically the most plausible, it is still necessary to further clarify potential contributions from other types of intermediates, such as radicals and their corresponding hydrogen bonding capabilities [57].

**Scheme 2:**
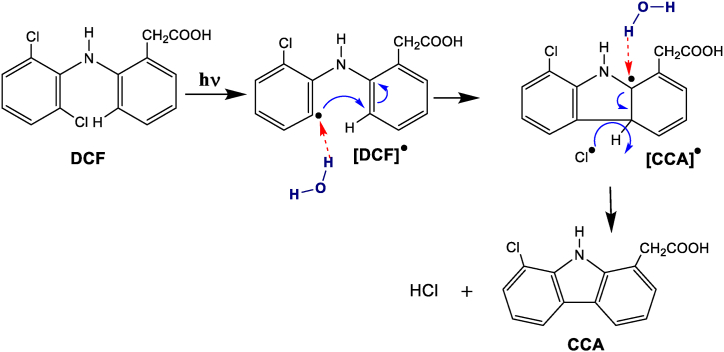
Proposed photoconversion pathway of DCF to CCA through free radicals intermediates. The red arrows refer to the HB between the intermediate free radicals and solvent molecules (water as an example).

As can be noted from most of the proposed mechanisms in the literature for the photocyclization of DPA and Ar-X, the reaction pathways include the formation of aryl free radicals. Henceforth, considering the importance of the HB donation capability of the solvent as experimentally observed, it is noteworthy to mention that the proposed mechanistic pathway illustrated in Scheme 2 is consistent with the previously reported significance of the formation of HB between the aryl or alkyl free radicals and with solvent molecules [[58], [59], [60], [61]]. In a recent study, Yang et al. investigated the role of chlorine radicals on the photoinduced alkane oxidation reaction, for which the formation of radical intermediates is associated with the elimination of hydrogen chloride through radials' reaction [60]. In their study, they reported that the methyl radicals’ intermediates are stabilized by hydrogen bonding with the solvent molecules (alcohols). In light of this, we hypothesize a mechanistic pathway for the photoconversion of DCF, as displayed in Scheme 2. Scheme 2 displays the general hypothesized pathway for the photoconversion of DCF into CCA through the route of formation of DCF and CCA free radicals. The irradiation of DCF leads to the formation of a DCF radical (DCF^●)^ that is stabilized by the formation of a complex adduct with the solvent molecules through hydrogen bonding, where the roles of DCF^●^ and the solvent molecule is such complexation are hydrogen bonding acceptor and donor, respectively. In accordance with the experimental findings, it can be suggested that the stabilization of DCF and CCA radicals can be enhanced by solvent molecules with increasing acidity.

Furthermore, the apparent photoconversion rate (*ν*_app_) can be presumably governed by the following equation:

(4) $$\nu_{app}=\nu_{ISC}+\nu_{f}+\nu_{cyc}$$

where ν_*ISC*_, ν_*f*_, and ν_*cyc*_ correspond to the rates of intersystem crossing from the first excited singlet state to the first excited triplet state, the formation of DCF^●^ and Cl^●^ radicals, and the cyclization step, respectively. The poor fluorescence properties of DCF can be attributed to ultrafast intersystem crossing (high *k*_*ISC*_), a property that exhibits no enhancement upon changing the solvent polarity or hydrogen-bonding capability. Therefore, the observed influence of the solvent on the photochemical conversion of DCF is likely due to the effect the solvent had on the rates of radical production and cyclization processes. In our previously reported computational results regarding the photocyclization of DCF, we demonstrated that DCF^●^ can form complexes with explicit water molecules via both halogen- and hydrogen-bonding [55]. The computationally optimized geometry revealed non-planar and planar structures for DCF and CCA, respectively. Consequently, due to the nonplanar structure of DCF, the photocyclization pathway of DCF requires a significant step of intramolecular rotation to facilitate ring closure. This rotation is necessary to generate the planar carbazole derivative. As such, the stability of HB complexes of DCF^●^ and CCA^●^ with solvent molecules may be the key factor in the effect of solvent molecules. One can argue that stabilization of DCF^●^ by protic polar solvents via HB can decrease the rate of photoconversion. However, the MLRA results revealed that protic solvents could increase the corresponding rate in terms of solvent acidity. In view of that, it can be hypothesized that the HB complexes of CCA^●^ may exhibit a more dominant factor compared to their DCF^●^ analogues. This may be interpreted in terms of structure planarity, where the planar structure of CCA^●^ permits enhanced accessibility for the solvent molecules compared to DCF^●^ to form stable HB complexes, and hence the step DCF^●^ → CCA^●^ is crucial for enhancing the overall rate of DCF photoconversion, for which the solvent acidity plays an important role.

## 4 Concluding remarks

Steady-state fluorescence spectroscopy measurements were successfully used to explore the effects of the polarity and hydrogen-bonding capability of a solvent on the photoconversion of DCF. The common generalized technique of Kamlet–Taft was employed to correlate the experimental kinetics data, specifically the reaction rate, which exhibited notable advantageous applicability over the Lippert–Mataga technique, as it takes into consideration the effect of a solvent's hydrogen-bonding capability simultaneously with the polarity effects. Interestingly, we showed that not only did the rate of the DCF photocyclization process depend on the polarity of the solvent, but the solvent's ability to form hydrogen bonds was the main factor in the overall rate. The study specifically revealed that the rate of DCF photocyclization is influenced by the polarity of the solvent, with higher polarity leading to an increased rate. Moreover, the rate of photocyclization is significantly enhanced with solvents that have a higher hydrogen-bond-donating capacity (acidity), while it is slowed down to a lesser extent with solvents that have a higher hydrogen-bond-accepting capability (basicity). These findings contribute to our comprehension of DCF photoconversion, which is crucial for explaining the photochemical behavior of significant biomedical materials.

## CRediT authorship contribution statement

**Abdulilah Dawoud Bani-Yaseen:** Conceptualization, Data curation, Formal analysis, Investigation, Methodology, Project administration, Resources, Supervision, Validation, Writing – original draft. **Raed M. Al-Zoubi:** Validation, Writing – review & editing. **Mohanad Shkoor:** Validation, Writing – review & editing.

## Declaration of competing interest

The authors declare that they have no known competing financial interests or personal relationships that could have appeared to influence the work reported in this paper.
