# Supplementary material for: Spectrofluorometric investigations on the solvent effects on the photocyclization reaction of diclofenac
Source: Heliyon. 2023 Oct 11;9(11):e20767. doi: 10.1016/j.heliyon.2023.e20767 (PMC10618426; doi:10.1016/j.heliyon.2023.e20767)
Supplement: Multimedia component 1 [file mmc1.docx]

***Supplementary Data***

**Spectrofluorometric Investigations on the Solvent Effects on the Photocyclization Reaction of Diclofenac**


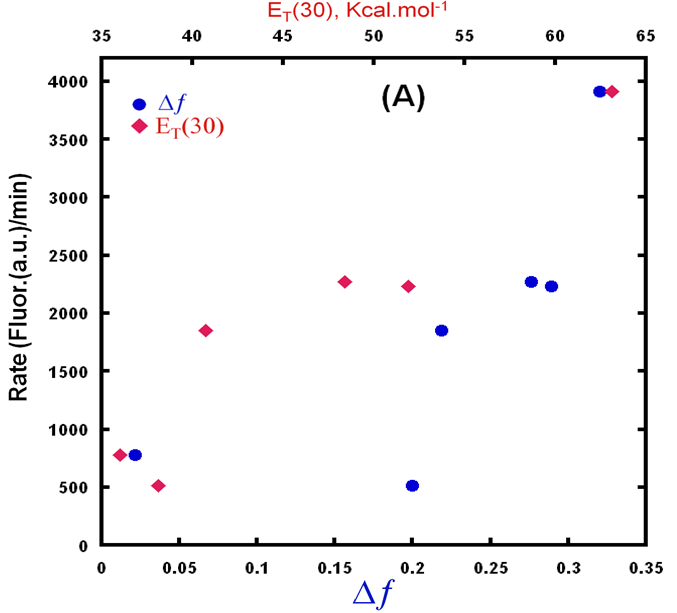


**Figure S1:** Variation in the rate of DCF photochemical reaction as a function of solvent polarizability (Δ*f*) and empirical polarity parameter E_T_(30).

**
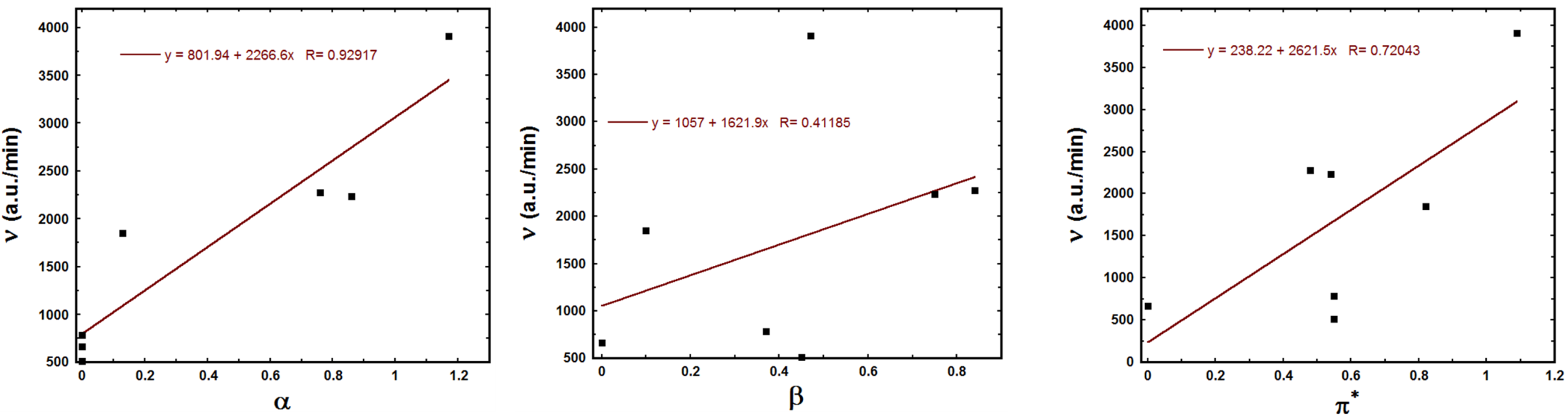
**

**Figure S2:** Variation in the rate of DCF photochemical reaction as a function of and the solvent hydrogen-bond donor (α) and acceptor (β) capabilities, and the solvent polarizability(π*).
